# E-Commerce Shopping Motivation and the Influence of Persuasive Strategies

**DOI:** 10.3389/frai.2020.00067

**Published:** 2020-11-23

**Authors:** Ifeoma Adaji, Kiemute Oyibo, Julita Vassileva

**Affiliations:** Department of Computer Science, University of Saskatchewan, Saskatoon, SK, Canada

**Keywords:** persuasion, shopping motivation, e-commerce, shopper typology, persuasive strategies

## Abstract

Persuasive strategies are used to influence the behavior or attitude of people without coercion and are commonly used in online systems such as e-commerce systems. However, in order to make persuasive strategies more effective, research suggests that they should be tailored to groups of similar individuals. Research in the traits that are effective in tailoring or personalizing persuasive strategies is an ongoing research area. In the present study, we propose the use of shoppers' online shopping motivation in tailoring six commonly used influence strategies: *scarcity, authority, consensus, liking, reciprocity*, and *commitment*. We aim to identify how these influence strategies can be tailored or personalized to e-commerce shoppers based on the online consumers' motivation when shopping. To achieve this, a research model was developed using Partial Least Squares-Structural Equation Modeling (PLS-SEM) and tested by conducting a study of 226 online shoppers. The result of our structural model suggests that persuasive strategies can influence e-commerce shoppers in various ways depending on the shopping motivation of the shopper. *Balanced buyers*—the shoppers who typically plan their shopping ahead and are influenced by the desire to search for information online—have the strongest influence on *commitment* strategy and have insignificant effects on the other strategies. *Convenience shoppers*—those motivated to shop online because of convenience—have the strongest influence on *scarcity*, while *store-oriented shoppers*—those who are motivated by the need for social interaction and immediate possession of goods—have the strongest influence on *consensus*. *Variety seekers*—consumers who are motivated to shop online because of the opportunity to search through a variety of products and brands, on the other hand, have the strongest influence on *authority*.

## Introduction

Simply selling products online is no longer sufficient for e-businesses to differentiate themselves from their online competitors. With many more companies now having an online presence, companies are seeking new ways to outdo their competitors. Businesses have to come up with new strategies to influence the purchasing decision of their clients.

Persuasion and how it is used to influence people's attitudes and the way they behave are an active research area in several domains including e-commerce. Persuasion is the use of influence strategies to change how people act and behave without coercion (Fogg, 2002). These strategies are often referred to as persuasive strategies (Fogg, 2002) and are implemented in various forms such as messages targeted at an audience. For example, some e-commerce companies use phrases such as “*Only a few left in stock*” to show that some products are in limited quantity. Existing research indicates that the use of persuasive strategies are more likely to result in a desired attitude or behavior change when these strategies are tailored to an individual or a group of individuals who are similar (Kaptein, 2011; Kaptein et al., 2012, 2015; Orji et al., 2014b).

Current efforts at tailoring persuasive strategies have used factors such as users' personality traits (Hirsh et al., 2012) and demographic data of users such as age (Phillips and Stanton, 2004), gender (Orji, 2016), and culture (Kramer and Spolter-Weisfeld, 2007). Despite the success in the use of personality, age, gender, and culture in tailoring persuasive strategies, in cases where these consumer characteristics are not known, such as in e-commerce, using these traits to tailor persuasive strategies is not possible. Therefore, for influence strategies to be personalized in online commerce, it is important to determine what other traits can be used to tailor persuasive strategies to individual users or groups of similar users to make them effective in bringing about a behavior or attitude change. We aim to fill this gap in the current paper by identifying if other factors such as a consumers' shopping motivation can be effectively used to tailor influence strategies to the consumers.

Research in e-commerce suggests that the intention of shoppers to buy a product is can be predicted by their motivation for shopping (Pappas et al., 2017). While shopping, online shoppers are not influenced the same way and thus, do not act the same way in terms of their shopping patterns and behaviors (Ganesh et al., 2010). Thus, in order to create a tailored or personalized online shopping experience for a shopper, it is essential to identify the factors that influence them (Pappas et al., 2017). Several typologies of shopping motivation exist. One such typology is that of Rohm and Swaminathan (2004), which classifies consumers into four categories according to their motivation for shopping online: *convenience shopper, store-oriented shopper, balanced buyer*, and *variety seeker*. We chose to use this typology in this study because of its popularity and widespread usage in e-commerce research (Ganesh et al., 2010; Pappas et al., 2017). Being able to identify what persuasive strategy each shopper type is influenced by could result in a shopping experience that is more personalized to the consumer. For instance, if *variety seekers* are influenced by *consensus* (looking to others who are similar to themselves in uncertainties) using messages that show consensus, for example, what products similar people have bought in the past, could influence this set of shoppers to buy particular products.

The aim of this paper is to identify what persuasive strategies e-consumers are influenced by based on their shopping motivation. To accomplish this, we conducted a study of 226 e-commerce shoppers to explore how the various shopper types (which are based on shopping motivation) are influenced by persuasive strategies. We measured persuasive strategies using Cialdini's six influence strategies (Cialdini, 2009) because they are commonly used in several domains including e-commerce (Kaptein and Parvinen, 2015). We developed a path model using partial least squares structural equation modeling (PLS-SEM) and tested it using the data from the survey. The result of our analysis suggests significant differences in the susceptibility of the various shopper types to the different influence strategies. In particular, *balanced buyers* were most highly influenced by *commitment* and were insignificantly affected by the other strategies. This suggests that *balanced buyers* are more likely susceptible to *commitment* strategy; thus, if they commit to purchasing a product, they will likely do so. Also, *convenience shoppers* were more influenced by *scarcity* compared to the other strategies, while *store-oriented shoppers* were more influenced by *consensus* compared to other strategies. Furthermore, *variety seekers* were more influenced by *authority* compared to other strategies. Possible guidelines in implementing these persuasive strategies in e-commerce are suggested.

## Related Work

### Shopping Motivation

Research has shown that products can be effectively tailored to the various segments of consumers by classifying the customers according to how they are motivated to shop online (Rohm and Swaminathan, 2004). In addition, classifying consumers based on their motivation informs businesses of what clients look out for and their attitude during the shopping decision-making process (Keng Kau et al., 2003).

There are various taxonomies of online shoppers such as the typology of Keng Kau et al. (2003). They categorize e-commerce shoppers into six groups based on the information-seeking patterns of consumers in addition to their online motivation and concerns during the shopping process. Another popular typology is that of Rohm and Swaminathan (2004), who categorize online shoppers into four groups: *variety seekers, convenience shoppers, store-oriented shoppers*, and *balanced buyers* according to the shopping motivation of the consumers. According to the authors, the online convenience of shopping and the ability to save time and effort motivate *convenience shoppers* to shop online. This category of e-consumers, however, is not motivated to immediately acquire the products they buy. The possibility of searching for different brands and products from several stores motivates the *variety seekers*. Being able to explore product details online as the *variety seekers* motivates the *balanced buyers*. However, the *balanced buyers* differ from the *variety seekers* because the *balanced buyers* typically plan their purchases ahead, unlike the *variety seekers*, who do not. Social interaction motivates the *store-oriented shoppers*, in addition to the desire to acquire the purchased goods immediately.

The online clickstream data of consumers can be used to identify the various categories of shoppers. *Variety seekers*, for instance, compare different stores, products, and brands while shopping because they seek variety (Rohm and Swaminathan, 2004). *Variety seekers* will likely spend more time reviewing and comparing prices, promotions, brands, and the features of products before making a purchase decision (Keng Kau et al., 2003). Thus, if consumers' online click activity is analyzed, their browsing pattern can show if they are searching for a variety of products and if they can be classified as *variety seekers*. The *store-oriented shoppers* seek social interaction (Rohm and Swaminathan, 2004) and thus will likely engage in interaction or dialogue with other consumers on the e-commerce platform before making a purchase. Interaction in e-commerce is usually by asking other customers questions about the products they have previously purchased (Adaji and Vassileva, 2017) or by interacting with a site's chatbot if one exists. Thus, shoppers who typically interact with other consumers or with the site's chat agent before making purchases could be identified as *store-oriented shoppers*. In addition, because *store-oriented shoppers* are influenced to possess their products immediately (Rohm and Swaminathan, 2004), this category of shoppers will likely pay for express delivery of their products while other categories of shoppers will not. The online convenience of shopping and the ability to save time and effort motivate *convenience shoppers* to shop online (Rohm and Swaminathan, 2004). This category of consumers shops online for specific products and services; they do not seek variety across several channels but are motivated by the convenience of online shopping, effort, and time saving (Rohm and Swaminathan, 2004). Therefore, it is likely that *convenience shoppers* will not spend time and effort browsing different brands as the *variety seekers* would likely do. Their clickstream data could reveal their browsing patterns. Also, because social interaction does not influence *convenience shoppers*, this category of consumers may not participate on an e-commerce website's social platform, where questions are asked and answered and reviews posted. Furthermore, since *convenience shoppers* are not influenced to acquire purchased products immediately, they may be unwilling to pay extra for the express delivery of their products.

In the current paper, the typology of Rohm and Swaminathan (2004) was used because the four classes of shoppers are based on online shopping behavior and they have several similarities to other existing typologies, such as (Keng Kau et al., 2003; Moe, 2003). In addition, as far as we know, no other study exists that uses this popular typology in tailoring influence strategies in e-commerce.

### Persuasive Strategies

According to Simons and Jones (2011) persuasion is “human communication designed to influence the autonomous judgments and actions of others.” Persuasion attempts to change the way people think or act without being forced or coerced. Usually, with persuasion, the person being persuaded is in charge of the final decision of whether to change their behavior (Simons and Jones, 2011). Persuasive strategies are the different methods with which persuasion is implemented. Several taxonomies of persuasive strategies exist. The Persuasive Systems Design framework (PSD) (Oinas-Kukkonen and Harjumaa, 2008) consists of 24 persuasive strategies that the authors recommend for the design and development of persuasive systems. These are classified into four categories, defined by the task the strategy is intended to accomplish: primary task support, dialogue support, social support, and system credibility support. The categories of the PSD framework and the persuasive strategies that fall within each category are shown in Table 1.

**Table 1:** Categories and persuasive strategies of the PSD framework.

**Primary task support**	**Social support**	**System credibility support**	**Dialogue support**
Reduction	Social learning	Trustworthiness	Praise
Tunneling	Social comparison	Expertise	Rewards
Tailoring	Normative influence	Surface credibility	Reminders
Personalization	Social facilitation	Real-world feel	Suggestion
Self-Monitoring	Cooperation	Authority	Similarity
Simulation	Competition	Third-party endorsement	Liking
Rehearsal	Recognition	Verifiability	Social role

The persuasive strategies of the PSD framework are commonly used in e-commerce systems to influence the shopping behavior of consumers. For example, amazon.com implements the 1-Click feature, which makes it easier for consumers to purchase items without having to go through the longer process of adding the item to their cart, filling out their shipping and payment details, and then placing the order for the product (Adaji and Vassileva, 2016). This significantly *reduces* the time it takes for a shopper to make a purchase. In addition, amazon.com allows its consumers to *self-monitor* their activities by providing a way for them to check the status of their orders and any previous purchases that they have made (Adaji and Vassileva, 2016). The online store childrensplace.com *suggests* other items to shoppers using the phrase “We think you'll also like” and images of suggested products. Walmart.ca influences people to shop by allowing them *learn* from others through the use of the “Questions and Answers” platform on the site.

Another common taxonomy of persuasive strategies is the six influence strategies of Cialdini which include *reciprocity, scarcity, commitment, authority, consensus, and liking* (Cialdini, 2009). *Reciprocity* is based on most people's need to always return a favor or repay in kind. An example of reciprocity is when an online bookstore offers its customers free e-books which could lead to more purchases from these customers because they feel the need to “return the favor^1^” A second example of *reciprocity* is the use of loyalty rewards programs offered by different companies. In their study of understanding customer retention and value based on their membership of a loyalty program, Bolton and Kannan (2000) conducted a study on a rewards-for-usage program offered by a financial services company. The company allows its members to accumulate points when they make purchases with their bank cards, which are redeemable through different stores offering a variety of products and services. The authors posit that the customers who benefited from the loyalty reward program were more likely to overlook the negative evaluations of the company because these customers believe they are receiving good value for their money in the form of the rewards program.

Because humans are typically consistent in nature, when they commit to carry out a particular action, they usually do so. The *commitment* persuasive strategy suggests that if a system can get people to commit to a particular behavior, because of the consistent nature of humans, they likely carry out the target behavior (Cialdini, 2009). This strategy hinges on the theory of Cognitive Consistency, which suggests that because inconsistencies that are internal result in a state of tension in people, when faced with such internal inconsistencies, people behave in ways that could lower them (Feldman, 2013). Therefore, humans are commonly consistent in nature. In order to influence shoppers to commit to shopping with them, e-stores such as amazon.com offer consumers the opportunity to add products to a *wish list* (Kaptein, 2011). The clothing store childrensplace.com uses the foot in the door technique (Freedman and Fraser, 1966) by offering shoppers a discount on their next purchase.

The *consensus* persuasive strategy (also known as *social proof*) (Cialdini, 2009) proposes that people often took up to other people that they are similar to when not sure about how to behave and act. A common method of implementing *consensus* in e-commerce is by using the feature “customers who bought this item also bought,” which displays products similar to that being viewed by a client. This feature is used on various e-commerce sites such as amazon.com, walmart.ca and realcanadiansuperstore.ca. Some online stores implement consensus by showing shoppers the number of people who have purchased a product (Kaptein, 2011).

According to Cialdini (2009), humans tend to believe and obey authority figures; therefore, when people decide what behavior to adopt in a given situation, the presence of authority figures can influence people's decisions. Authority figures include experts in a field, one's boss, or religious leaders (Cialdini, 2001, 2009). The endorsement of influencers and reviews from experts in a field are some ways that e-commerce companies implement *authority* (Kaptein, 2011).

Cialdini (2009) suggests that most times, people are more influenced by something or someone that they like; this describes the *liking* persuasive strategy. Therefore, if someone that a person likes makes a request, they are more likely to fulfill the request compared to a request from someone that the person does not like. Online consumers usually shop with companies that they like based on the recommendations and personalization that they receive from such companies (Li et al., 2013).

The *scarcity* principle, according to Cialdini (2009), is “the rule of the few.” The author posits that humans crave for items that are limited and not readily available because scarce items are often considered more valuable than items that are abundant. In implementing scarcity, Cialdini suggests that businesses should highlight the unique benefits of a product, its exclusivity, and what people may lose by not purchasing a product (Cialdini, 2001). E-commerce vendors implement this strategy by announcing special limited time offers to their clients (Kaptein, 2011). Amazon.com implements scarcity by stating when a product is limited in stock or edition, with phrases like “only three left in stock.” Laura.ca, a Canadian clothing retailer, uses the phrase “Hurry, *n* item(s) left for delivery,” (where *n* represents a low number) in pink background to indicate a product is limited in stock. Walmart.ca uses the phrase “Almost sold out” in a red font to indicate items that are limited in quantity.

The use of Cialdini's six persuasive strategies to influence behavior change is an active research area. In their research on the effect of heterogeneity in persuasion in online systems, Kaptein and Eckles (2012) investigated three of Cialdini's six influence strategies: consensus, authority, and scarcity. Using product evaluations, the authors explored how the three persuasive strategies influence people differently. The authors concluded that, compared with a tailored approach, a one-size-fits-all method was less effective in influencing people to adopt a given behavior. In other words, the authors showed significant differences in the average effects of the three persuasive strategies. For example, some participants that were positively influenced by *consensus* were negatively influenced by *authority*. In addition, the authors suggested that using the wrong influence strategy could result in negative effects in terms of behavior change compared with using no strategy at all. Furthermore, using the best persuasive strategy for a person or similar individuals could influence them to carry out the desired change in attitude or behavior compared to using the best average strategy.

We chose to use Cialdini's six persuasive strategies in this study because they are popularly used in consumer studies research. In addition, compared with the PSD framework where some strategies are very similar to others (for example, simulation and rehearsal), the six strategies of Cialdini are very distinct and different from each other. Furthermore, there is currently no existing study that maps shoppers' online motivation to the persuasive strategies they are influenced by using Cialdini's strategies.

### Tailoring Persuasive Strategies

Previous studies have shown that tailored persuasive strategies are more likely to bring about the desired behavior change compared to non-tailored strategies. For example, in their study of adaptive persuasive messages in e-commerce, Kaptein (2011) concluded that significant individual differences exist in users' responses to the implementation of various persuasive strategies. Similarly, in their study of influencing different gamer types, Orji et al. (2014b) determined that different gamer types are influenced by different persuasive strategies; the gamer type *achiever* is significantly influenced by *cooperation*, while a *daredevil* is influenced by *simulation*. Furthermore, Kaptein et al. (2012) studied the use of persuasive strategies in the form of messages to curtail snacking, and concluded that their study participants who received tailored messages significantly reduced their snacking consumption compared with the participants who did not. These results suggest that a one-size-fits-all approach to the implementation of persuasion will not likely bring about the desired behavior or attitude change among the users of a system.

Several factors have been used to tailor persuasive strategies. The use of personality traits is one of such factors. Hirsh et al. (2012), in their study of personalized persuasion, tailored persuasive messages for a single product via advertisements to shoppers based on their Big Five personality traits. The authors concluded that when persuasive messages are tailored to personality traits, it increased the impact of the messages. In their study of tailoring persuasion, Smith et al. (2016) tailored persuasive reminders to participants based on their personalities. The authors found significant differences in participants' preferences to the persuasive messages based on the participants' personalities. Alkiş and Taşkaya Temizel (2015) similarly researched the effect of tailoring influence strategies based on people's personalities. Their study of university students using the Big Five personality traits concluded that there were major differences in the influence of personality traits on influence strategies and thus that personality is a good factor in tailoring persuasive strategies.

The demographic data of users, such as age, gender, and culture, have also been used in personalizing influence strategies. In their study of motivational text messages, de Vries et al. (2017) concluded that gender influences the perception of motivational messages, thus, it can be used to tailor messages to people when the gender is known. Busch et al. (2016) investigated the role of gender in the persuasiveness of influence strategies. The authors concluded that different genders were influenced differently. Similarly, Orji et al. (2014a) examined the role of gender in the persuasiveness of influence strategies. The authors also concluded that males and females are influenced differently. Kramer and Spolter-Weisfeld (2007) researched the effect of the use of culture to tailor persuasive messages. Their results suggest that the cultural orientation of consumers significantly influenced their reception of personalized messages. The authors concluded that consumers, based on their culture—individualistic or collectivistic—responded differently to persuasive strategies. For example, collectivists were receptive to non-tailored recommendations, compared with individualists, who were not. Similarly, in her study of how the different cultures are influenced by persuasive strategies, Orji (2016) suggests that participants were influenced differently based on their culture, collectivistic or individualistic. Orji concluded that while collectivists were influenced by *reciprocity, authority, consensus*, and *liking*, individualists were not. Furthermore, Phillips and Stanton (2004) investigated age-related differences in persuasion and concluded that there are significant distinctions in the influence of persuasive strategies according to age. According to the authors, while younger consumers will likely recall information presented in ads, they will less likely be persuaded by it. On the other hand, older consumers will less likely recall information on ads but will more likely be persuaded by it.

In systems where these factors are not known, such as in e-commerce, it becomes difficult to tailor persuasive strategies to users to make these strategies more effective in bringing about the desired behavior change. For example, most e-commerce companies do not ask the gender or age of their clients during checkout. In addition, e-businesses make it possible for one to shop as a visitor without having to register an account with the merchant. Furthermore, people often shop for others, thus making it impossible to determine the gender of a shopper based on the content of their shopping cart. This study aims to fill this gap by using shoppers' online motivation instead of demographic data of shoppers to tailor persuasive strategies. There is currently no study that has done this to the best of our knowledge.

### Other Factors That Influence Shopping Motivation

This paper focuses on the influence of persuasive strategies on shopping motivation, in particular, how different shoppers are influenced. We, however, recognize that other factors influence consumers' shopping motivation, such as the shopping value derived from the shopping experience. Value proposition has two popular dimensions: utilitarian and hedonic values. Consumers who possess high hedonic shopping value typically buy products for the happiness or pleasure that they get while shopping and not for how useful the product or service is (Overby and Lee, 2006; Bridges and Renée, 2008). Shoppers in this category are usually spontaneous, motivated to avoid pain, and drawn to pleasure (Babin et al., 1994; O'Shaughnessy and Jackson O'Shaughnessy, 2002).

Hedonic and utilitarian shopping values are an active research area in e-commerce. In their study of e-commerce consumers' purchase and shopping well-being, Yu et al. (2018) investigated the role of hedonic and utilitarian shopping values on the intention of consumers to purchase in shopping carnivals held online. The authors concluded that people with hedonic shopping values are persuaded by entertainment while those with utilitarian shopping values are influenced by saving money, selection, and convenience. Yu et al.'s study differs from that presented in the current paper because while the authors investigated shopping motivation in the form of hedonic and utilitarian shopping values while we investigated shopping motivation in the form of different shopper types.

Adaji et al. (2019) also researched the effect of influence strategies on the shopping motivation of online consumers based on their shopping value. The authors defined shopping motivation based on the value (hedonic or utilitarian) that shoppers derived while shopping. The authors suggest that people with high hedonic value are persuaded to purchase scarce and limited products while people with high utilitarian shopping value are influenced by their social circles. The present study differs from that of Adaji et al. because the authors defined shopping motivation based on the hedonic and utilitarian shopping values of consumers but the present study defines shopping motivation based on the shopper type taxonomy of Rohm and Swaminathan (2004). To the best of our knowledge, this has not been done before.

## Research Design and Methodology

The research question, design and methods used in addressing the research question are presented in this section.

### Research Question

The overarching research question that is addressed by this paper is the following:

How are e-commerce shoppers influenced by persuasive strategies based on their different motivations to shop online?

### Methodology: Structural Measurement Model

**Figure 1:**
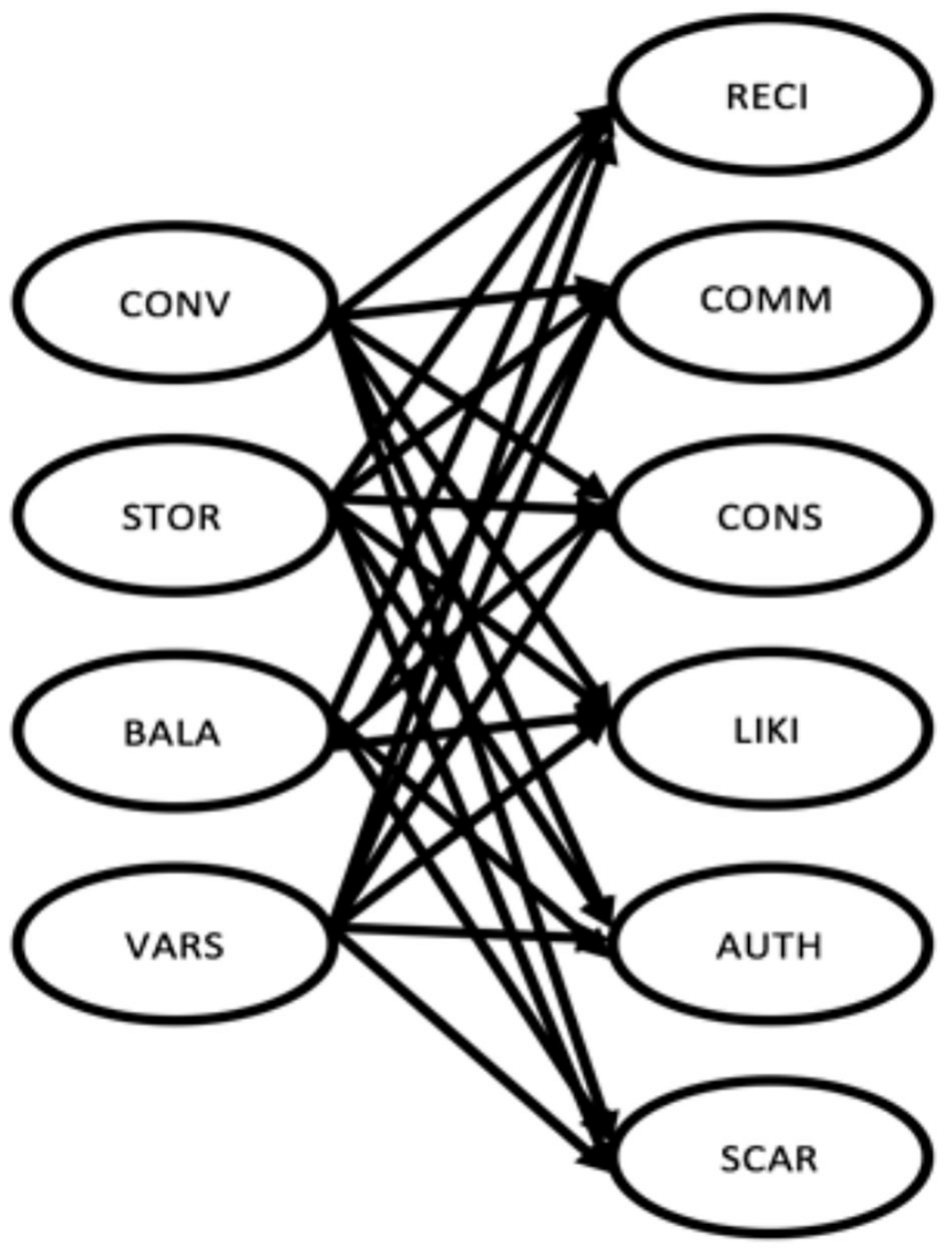
Research model with all paths assumed to be positive. STOR, store-oriented shopper; CONV, convenience shopper; VARS, variety seeker; BALA, balanced buyer; RECI, reciprocity; COMM, commitment; CONS, consensus; LIKI, liking; AUTH, authority; SCAR, scarcity.

To answer our research question, we developed a path model (shown in Figure 1) using PLS-SEM to measure the susceptibility of the four shopper types (based on online shopping motivation: *variety seekers, convenience shoppers, store-oriented shoppers, and balanced buyers*) to Cialdini's (2009) six influence strategies: *scarcity, consensus, authority, commitment, reciprocation, and liking*. The model was developed using four constructs to represent the four shopper types and six constructs to represent the six persuasive strategies. As defined by the research question, the aim of the model is to measure the influence of the different persuasive strategies on the shopper types—in other words, to determine which persuasive strategy has the highest influence on the different shopper types.

Rohm's scale which consists of four constructs and 17 questions was used to measure shopping motivation (Rohm and Swaminathan, 2004). The susceptibility to persuasive strategies was measured using the *susceptibility to persuasive strategies* scale of Kaptein et al. (2009), which is made up of six constructs and 32 questions.

In carrying out the PLS-SEM, bootstrapping was implemented using a random sample size of 5,000 (with replacement) to derive the distribution to be used in the model for the different constructs as suggested by Hair et al. (2016). Also, we determined the indicator reliability of our model, its internal consistency reliability, the convergent validity and discriminant validity to ensure they met the minimum requirements as required in PLS-SEM analysis (Hair et al., 2016). These results are presented in section Evaluation of Global Measurements. The path coefficient, β, between constructs was also computed.

To test the model, we created a survey online using the instruments mentioned above. We measured all items on a seven-point Likert scale, where 1 was strongly disagree, and 7 was strongly agree.

### Participants

We carried out a study of e-commerce shoppers to test our model. The questions were presented in an online survey. In all, 226 e-commerce shoppers were recruited to take part in the study. Recruitment was done using Amazon's Mechanical Turk (AMT). In addition, we recruited some participants through various online social media and the news board of our University. We used AMT because it allows one to recruit a diverse set of participants, and it is an accepted method of recruiting participants (Hirsh et al., 2012; Jia et al., 2016). We have successfully used online social media and news boards in the past with success (Busch et al., 2016). Therefore, we used them again to recruit participants in this study. Participants were asked to answer the questions in the context of grocery shopping. The Behavioral Ethics Board of our University approved the study. The demographics of our participants are presented in Table 2.

**Table 2:** Participants' demographics.

**Demographics**	**Value**	**Frequency (%)**
Age	Below 30	55
	Between 30 and 49 inclusive	40
	Above 50	5
Gender	Female	44
	Male	56
Size of household	1–3 people inclusive	63
	4–5 people inclusive	34
	6 or more people	4
Combined Income of household	Below US$30,000	40
	Between US$30,000 and $75,000	42
	More than US$75,000	18
Origin/Continent	Europe	8
	Asia	35
	North America	48
	Others	9

## Data Analysis and Results

We analyzed the survey data using the SmartPLS tool^2^. SmartPLS is a commonly used tool for PLS-SEM and is popularly used in the research community because of its ease of use and ease of interpretation of results (Wong, 2013; Hair et al., 2016).

### Partial Least Squares Structural Equation Modeling (PLS-SEM)

PLS-SEM is used mainly in exploratory research to develop theories. It focuses on describing the variance of dependent variables in a research model. Even with a small sample size, PLS-SEM is known to achieve significant statistical results and does not require the distributional assumptions of other statistical methods (Hair et al., 2016). PLS-SEM does not rely on any distributional assumptions. Rather it uses bootstrapping to derive a distribution to be used in the model. In bootstrapping, subsamples are selected randomly and replaced from the original dataset. This goes on repeatedly until a substantial number of random samples have been created (Hair et al., 2016).

In carrying out the analysis of the structural model, bootstrapping was implemented with a random sample size of 5,000 (with replacement) as recommended by Hair et al. (2016).

### Evaluation of Global Measurements

Research (Hair et al., 2016) suggests that the relationship between indicators (which are measures of a construct or the questions asked for each construct) of each construct should be evaluated before the relationship between the constructs is considered. This is achieved by computing a model's internal consistency reliability, indicator reliability, convergent validity, and discriminant validity (Hair et al., 2016). The results of these measurements are presented in the following section.

#### Internal Consistency Reliability

The use of Cronbach's alpha in assessing internal consistency reliability is not recommended since it assumes that all the indicators of a construct are equally reliable (Hair et al., 2016). This does not always happen because oftentimes, the indicators of a construct do not have the same outer loadings. In addition, the number of items on a scale influences Cronbach's alpha; an increase in Cronbach's alpha often results from an increase in the number of items (Hair et al., 2016). A commonly used alternative for measuring internal consistency that, researchers suggest, is better than Cronbach's alpha is composite reliability (Wong, 2013; Hair et al., 2016). Composite reliability indicates whether the indicator variables (the questions asked for each construct) are a good measure of a construct. Table 3 shows that the composite reliability of all constructs is >0.6, the acceptable threshold (Hair et al., 2016). Therefore, we conclude that among all constructs, high levels of composite reliability were established.

**Table 3:** Composite reliability and AVE of constructs.

**Constructs**	**Composite reliability**	**Average variance extracted (AVE)**
Convenience shopper	0.875	0.637
Store oriented shopper	0.816	0.60
Balanced buyer	0.863	0.677
Variety seeker	0.638	0.50
Reciprocity	0.897	0.638
Scarcity	0.789	0.50
Authority	0.868	0.569
Commitment	0.832	0.50
Consensus	0.860	0.607
Liking	0.853	0.537

#### Convergent Validity

The degree of correlation between the indicators of a construct is referred to as the convergent validity. Because the indicators of a construct are alternatives to measuring the same construct, they should share a high variance. In structural equation modeling, the convergent validity of a model is often measured with the average variance extracted (AVE) (Wong, 2013; Hair et al., 2016). Table 3 shows that the constructs in the model have the minimum acceptable AVE values of at least 0.5 (Wong, 2013; Hair et al., 2016).

#### Indicator Reliability

Indicator reliability describes the size of the relationship between indicators that make up a construct and the construct (Hair et al., 2016). Research suggests that this relationship, known as the outer loadings, should be at least 0.4 for exploratory studies (Hulland, 1999; Wong, 2013; Hair et al., 2016). As shown in Table 4, the outer loadings in the model meet this criterion.

**Table 4:** Outer loadings of model.

	**Convenience shopper**	**Store-oriented shopper**	**Balanced buyer**	**Variety seeker**	**Reciprocity**	**Scarcity**	**Authority**	**Commitment**	**Consensus**	**Liking**
Convenience shopper 1	0.855									
Convenience shopper 2	0.853									
Convenience shopper 3	0.715									
Convenience shopper 4	0.759									
Store-oriented shopper 1		0.651								
Store-oriented shopper 2		0.806								
Store-oriented shopper 3		0.848								
Store-oriented shopper 4		0.721								
Balanced buyer 1			0.824							
Balanced buyer 2			0.810							
Balanced buyer 3			0.834							
Balanced buyer 4			0.808							
Variety seeker 1				0.643						
Variety seeker 2				0.726						
Variety seeker 3				0.771						
Variety seeker 4				0.698						
Variety seeker 5				0.701						
Reciprocity 1					0.814					
Reciprocity 2					0.846					
Reciprocity 3					0.860					
Reciprocity 4					0.670					
Reciprocity 5					0.785					
Scarcity 1						0.638				
Scarcity 2						0.768				
Scarcity 3						0.695				
Scarcity 4						0.769				
Scarcity 5						0.717				
Authority 1							0.715			
Authority 2							0.772			
Authority 3							0.833			
Authority 4							0.728			
Authority 5							0.715			
Commitment 1								0.695		
Commitment 2								0.683		
Commitment 3								0.634		
Commitment 4								0.788		
Commitment 5								0.724		
Commitment 6								0.788		
Consensus 1									0.727	
Consensus 2									0.735	
Consensus 3									0.798	
Consensus 4									0.806	
Consensus 5									0.775	
Consensus 6									0.703	
Liking 1										0.731
Liking 2										0.776
Liking 3										0.720
Liking 4										0.709

#### Discriminant Validity

Discriminant validity defines the extent to which a model's constructs differ from each other. Establishing discriminant validity indicates that each construct in the model is unique (Fornell and Larcker, 1981; Wong, 2013; Hair et al., 2016). If the square root of the AVE for each construct is higher than its highest correlation with other constructs, one can conclude that discriminant validity is established (Wong, 2013; Hair et al., 2016). As shown in Table 5, the square root of the AVE in bold is greater than the correlation values in each row. Therefore, we conclude that discriminant validity is established.

**Table 5:** Correlation of constructs.

**Constructs**	**Convenience shopper**	**Store-oriented shopper**	**Balanced buyer**	**Variety seeker**	**Reciprocity**	**Scarcity**	**Authority**	**Commitment**	**Consensus**	**Liking**
Convenience shopper	**0.798**									
Store-oriented shopper	−0.273	**0.775**								
Balanced buyer	0.287	0.089	**0.822**							
Variety seeker	0.165	0.180	0.243	**0.707**						
Reciprocity	0.295	0.076	0.251	0.262	**0.799**					
Scarcity	0.251	0.155	0.091	0.246	0.232	**0.707**				
Authority	0.223	0.149	0.245	0.345	0.522	0.338	**0.754**			
Commitment	0.300	0.085	0.431	0.281	0.589	0.256	0.513	**0.707**		
Consensus	0.076	0.269	0.038	0.265	0.298	0.264	0.429	0.264	**0.779**	
Liking	0.207	0.172	0.083	0.301	0.366	0.307	0.535	0.402	0.583	**0.733**

*The bold diagonal shows the square roots of AVE*.

### Structural Measurement Model: Evaluation

The structural model's results show the relationship between the independent variable and the dependent variable and how strong this relationship is. In addition, the results of the structural model describe how much the variances of the independent variables are defined by the dependent variables. This is represented by the path coefficients, β, between constructs. Table 6 shows the results of our structural model. The number of asterisks which range from 1 to 4 indicates how significant each direct path is. The asterisks represent the *p* < 0.05, < 0.01, < 0.001, and < 0.0001, respectively.

**Table 6:** Path coefficients of the structural model.

**Shopper types**	**Authority**	**Commitment**	**Consensus**	**Liking**	**Reciprocity**	**Scarcity**
Balanced buyer	0.116 n.s.	0.327****	−0.078 n.s.	−0.062n.s.	0.126 n.s.	−0.054 n.s.
Convenience shopper	0.186**	0.203**	0.138 n.s.	0.240**	0.259****	0.295*
Store-oriented shopper	0.142*	0.084 n.s.	0.276****	0.200*	0.105 n.s.	0.209*
Variety seeker	0.260**	0.153 n.s.	0.211***	0.240**	0.170*	0.173 n.s.

*N.s., not significant. The number of asterisks which range from 1 to 4 indicates how significant each direct path is. The asterisks represent the p-values < 0.05, < 0.01, < 0.001 and < 0.0001 respectively. The bold values show what persuasive strategy has the highest influence on the various shopper types*.

*Balanced buyer* is the most strongly affected by the strategy *commitment* (β = 0.327), and other strategies have insignificant effects. This suggests that *balanced buyers* are likely susceptible to a *commitment* strategy. *Convenience shopper* is the most strongly affected by *scarcity*, while *consensus* has the strongest effect on *store*-*oriented shopper*. In addition, *authority* has the strongest effect on v*ariety seeker*.

## Discussion

This study aims to identify what persuasive strategy each shopper type is influenced by. To answer the research question “How are e-commerce shoppers influenced by persuasive strategies based on their different motivations to shop online?” our results indicate that there are significant differences in the effects of various persuasive strategies on e-commerce shoppers as a result of their online shopping motivation. For example, while *balanced buyers* are influenced by *commitment* (β = 0.327)*, store-oriented shoppers* have the strongest susceptibility to *consensus* (β = 0.276).

### Balanced Buyers

The ability to search online for information motivates the *balanced buyers*, who are similar to the *variety seekers* (Rohm and Swaminathan, 2004). On the contrary, *balanced buyers* do not typically schedule their purchases in advance and are likely to make impulse purchases online (Rohm and Swaminathan, 2004). The results of this study suggest that *balanced buyers* are only influenced by the *commitment* strategy (β = 0.327). The *commitment* strategy (Cialdini, 2009) suggests that people are naturally consistent. Thus, if people commit to carrying out a target behavior, because of their consistent nature, they will likely carry out the behavior. Therefore, if an e-commerce site can get *balanced buyers* to commit to a particular behavior or action, this could result in this category of shoppers carrying out that behavior because they are influenced by *commitment*. This suggests that if *balanced buyers* commit to shopping for healthful meals, for example, they will likely do so.

**Figure 2:**
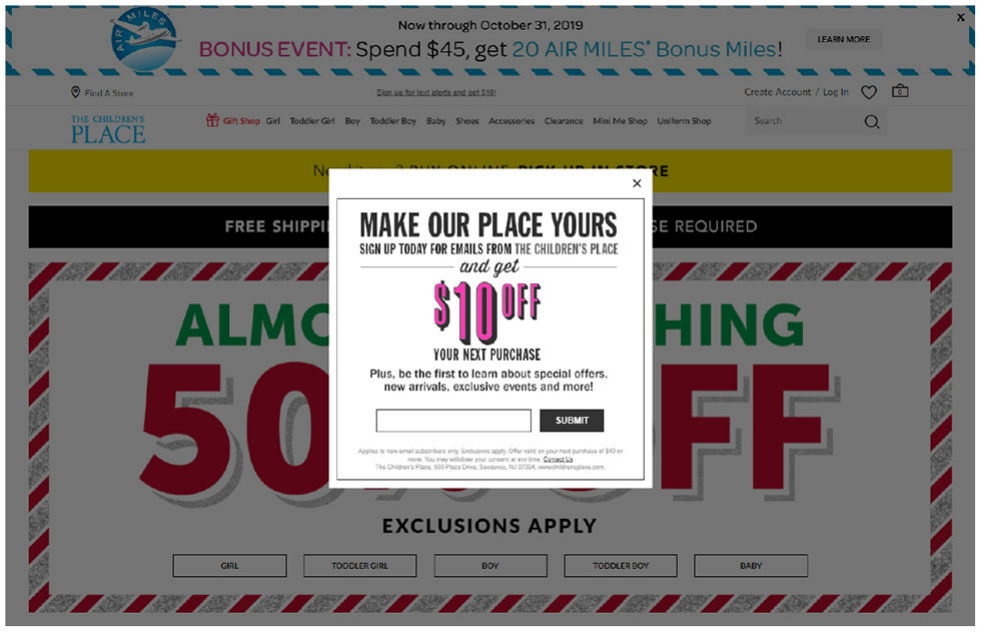
Example of the commitment strategy.

Cialdini (2001) suggests that a choice made explicitly, voluntarily, and publicly is more likely to change one's behavior compared to one made implicitly. An example of *commitment* is the “Foot in the door” technique (Freedman and Fraser, 1966). It suggests that if a person agrees to, and carries out a small request, it increases the likelihood that they will carry out a similar larger request. An example of implementing *commitment* in e-commerce is when an e-commerce company offers consumers a discount on their next purchase as shown in Figure 2.

This suggests that in tailoring persuasive strategies to shoppers in e-commerce, where the age, gender, and culture of shoppers are usually unknown, the shopping motivation of the consumer can be used.

### Convenience Shoppers

The minimal effort involved in online shopping, in addition to convenience and the time it saves compared to traditional shopping, motivates the c*onvenience shoppers* (Rohm and Swaminathan, 2004). These consumers do not expect to receive their goods immediately and are not motivated to carry out any social interaction while shopping. Furthermore, they do not search for a variety of products from different retailers (Rohm and Swaminathan, 2004). Our results suggest that *scarcity* (β = 0.295) has the strongest influence on *convenience shoppers*. Because this category of shoppers does not search for variety, it is not surprising that they are influenced by items that are limited.

**Figure 3:**
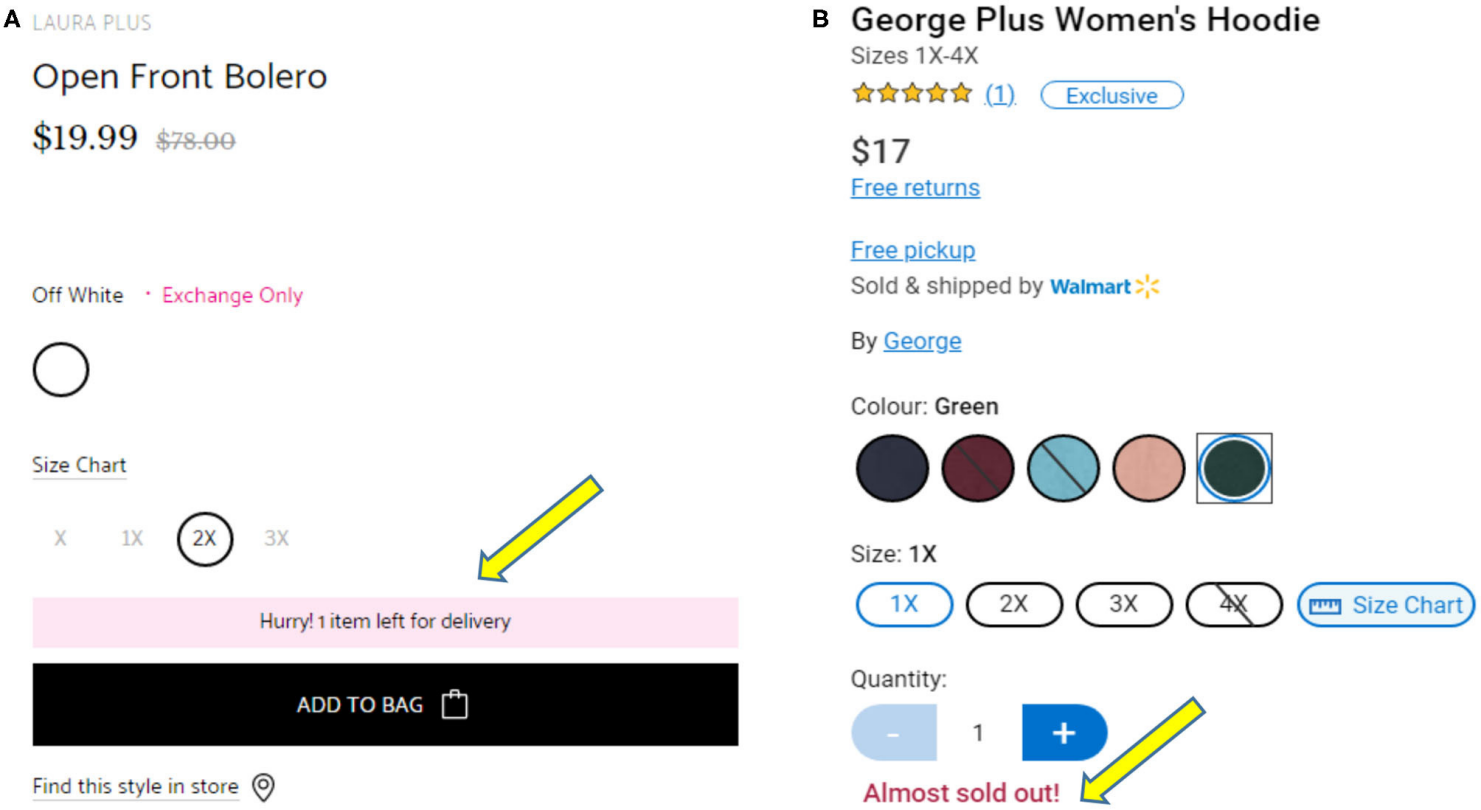
**(A)** Example of implementing scarcity in www.laura.ca. **(B)** Example of implementing scarcity in www.walmart.ca.

In implementing *scarcity*, Cialdini (2001) suggests that one highlight the unique benefits of an item and, in addition, state its exclusivity. E-commerce companies implement *scarcity* by stating when a product is *limited in stock*, is a *rare item*, or a *limited-edition* item. For example, Amazon^3^ uses the phrase “*n* items in stock” (where *n* represents a low number) when they are running out of an item. As shown in Figure 3A, Laura^4^, a popular clothing retailer in Canada, uses the phrase “Hurry, *n* item(s) left for delivery” (where *n* represents a low number) in pink background (indicated by the yellow arrow) when a product is limited in stock. Walmart^5^, a popular North American multinational corporation, uses the phrase “Almost sold out” in a red font as indicated by the yellow arrow in Figure 3B.

This result indicates that for shoppers in e-commerce, since the consumers' demographic data such as their age, gender, and culture are not known, their shopping motivation is a good factor in deciding how to tailor persuasive strategies.

### Store-Oriented Shoppers

**Figure 4:**
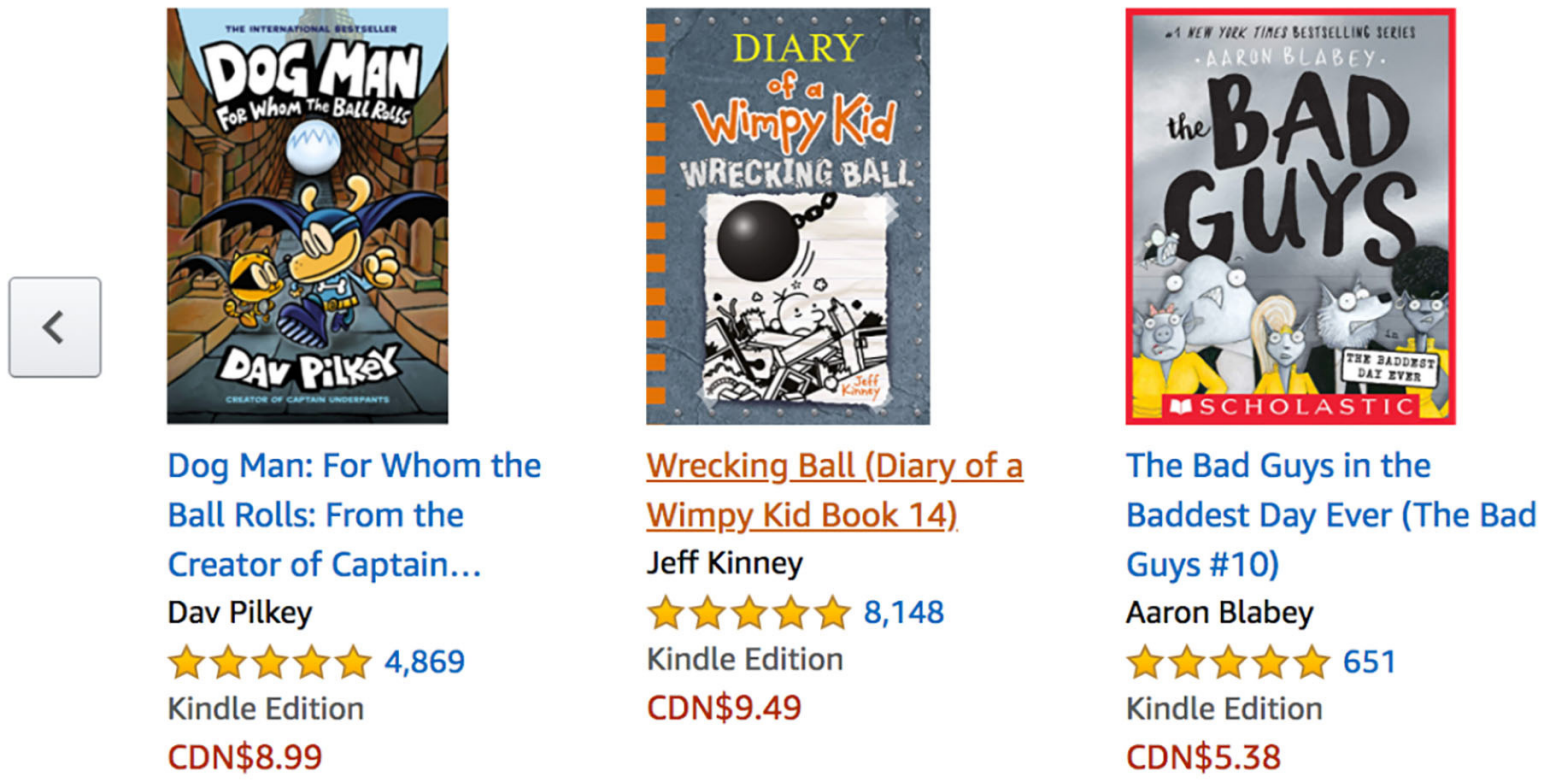
Example of implementing consensus on amazon.ca.

The desire to possess their products immediately and social interaction motivate *store-oriented* consumers to shop online (Rohm and Swaminathan, 2004). Our results suggest that this category of shoppers has the strongest influence on the persuasive strategy *consensus* (β = 0.276). *Consensus* (also referred to as social proof) implies that people look to others who are similar to them for suggestions on how to behave, especially when in doubt (Cialdini, 2001). This finding is reasonable because *store-oriented shoppers* are motivated to shop by social interaction. Thus, it is possible that they look to others for answers to questions about products and purchase decisions when they are shopping,

Cialdini (2001) suggests that in implementing the *consensus* strategy, one could use peer power whenever it is available. For example, he suggests that reviews from satisfied customers work better to influence prospective customers when the prospective client and satisfied client have something in common. One way to implement *consensus* in e-commerce is to show shoppers what products other consumers have bought or to show the products that are often purchased together. As shown in Figure 4, Amazon uses the phrase “Customers who read this also read” to show what books others have purchased based on the content of one's shopping cart.

In tailoring persuasive strategies to shoppers in e-commerce, this result shows that the shopping motivation of the consumer can be used.

### Variety Seekers

*Variety seekers* are motivated by the desire to seek a variety of products across various stores, product types, and brands (Rohm and Swaminathan, 2004). Our results suggest that this category of shoppers is most strongly influenced by the persuasive strategy *authority* (β = 0.260). This result is plausible because *variety seekers* who compare products across various channels will likely come across several reviews from *experts* who are knowledgeable about the product.

**Figure 5:**
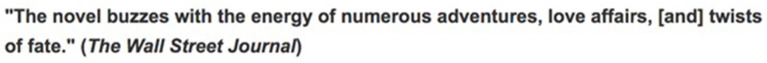
Example of implementing authority on amazon.ca.

The notion behind the *authority* strategy is that people listen to experts more than they listen to non-experts (Cialdini, 2001). Thus, claiming that a statement is one from experts could make people such as *variety seekers*, who are influenced by the *authority* strategy, change their attitude or behavior. Factors that can trigger the *authority* principle include (1) the use of titles such as Dr., Prof., CEO, (2) clothes such as religious outfits worn by priests, monks, and nuns, (3) status symbols such as an expensive car or suit (Cialdini, 2009), and (4) as well as quotes and endorsements from experts and authority figures. One way to implement *authority* while presenting a product to consumers is by using messages such as “The ministry of healthy suggests five daily servings of fruit” to influence consumers to purchase more fruit. Another example is to show reviews of people in authority such as book reviews of prominent authors or reviewers. As shown in Figure 5, Amazon includes book reviews from authority figures such as the Wall Street Journal.

Our results are an indication that the shopping motivation of consumers can be used as a factor in tailoring persuasive strategies to make them more effective in bringing about a change in attitude or behavior.

### The Strategies to Implement for the Various Shopper Types

The result shown in Table 6 indicates that *commitment* is the only strategy positively and significantly associated with *balanced buyers*. This suggests that consumers in this category will be significantly influenced only by *commitment*, making it the best strategy to implement for *balanced buyers*. *Convenience shoppers*, on the other hand, are influenced by all strategies except *consensus*, with *scarcity* having the strongest influence. *Store-oriented shoppers* are significantly influenced by *authority, consensus, liking*, and *scarcity*, with *consensus* having the strongest influence, while *variety seekers* are influenced by *authority, consensus, liking*, and *reciprocity*, with *authority* being the strongest.

### Best General Strategy for Shopper Types

For system designers who want to implement persuasive strategies based on the shopper types, if the designer's objective is an overall average effect across all shopper types, we recommend two strategies. The first recommended strategy is *liking*. Only two strategies, *liking* and *authority*, significantly influence three of the four shopper types. However, the influence on the shopper types of *liking* is stronger than the effect of *authority* for almost all the strategies. Therefore, *liking* is a better overall strategy to implement across all shopper types compared with *authority* or the other strategies. The second recommended strategy is *commitment*. No other strategy has an influence on *balanced buyers* except *commitment*. Thus, if a system designer is implementing strategies that will include all shopper types including *balanced buyers, commitment* has to be implemented in addition to *liking*.

If, on the other hand, the design objective is to maximize the effect of the persuasive strategy on the individual shopper types, the recommended strategies are *commitment, scarcity, consensus*, and *authority* for *balanced buyers, convenience shoppers, store-oriented shoppers*, and *variety seekers*, respectively.

### Limitations

This study is limited in a few ways. First, the results are self-reported and do not depend on the direct observation of participants. This is, however, common practice in consumer-based research as many successful studies in the past have been self-reported. Second, the sample size, 226, represents only a fraction of e-commerce shoppers worldwide. However, we are, of the opinion that with the thorough analysis we have carried out and the results obtained in this paper, the results would likely be similar if we had more participants.

## Conclusion and Future Work

Research suggests that influence strategies are effective in bringing about a change in people's attitudes and behavior. However, to make them effective, persuasive strategies should be tailored to people with similarities. In e-commerce, where the gender and age of shoppers are not known to the e-commerce vendor, there is a need to identify other traits that are effective in tailoring persuasive strategies to make them more effective in changing shoppers' attitudes or behavior. To fill this gap, this paper aimed to investigate how influence strategies could be tailored to e-commerce shoppers according to how they (consumers) are motivated to shop online. In particular, the paper aimed to answer the research question How are e-commerce shoppers influenced by persuasive strategies based on their different motivations to shop online? To achieve this, a structural model was developed using PLS-SEM and was evaluated by carrying out a study of 226 online shoppers.

Our results contribute and advance research in the area of e-commerce personalization and tailoring of persuasive strategies by showing that the different types of shoppers are significantly influenced by persuasive strategies differently. To answer our research question, different shopper types are influenced differently. Thus, a one-size-fits-all approach where the same persuasive strategies are applied to all types of shoppers will likely not be effective in changing shoppers' behavior. Rather, tailoring persuasive strategies to individual shopper types will result in the desired behavior change. In particular, the *commitment* strategy had the highest influence on the *balanced buyer* shopper type while the other strategies had insignificant effects. This indicates that *balanced buyers* are susceptible to *commitment* compared to other strategies. This implies that if balanced buyers can commit to making a purchase, they will likely carry it out. The “foot in the door” technique is one way that e-commerce companies can influence balanced buyers to commit to shopping with them by offering special discounts on their next purchase. *Convenience shoppers* had the highest influence on *scarcity*, which suggests that products that are labeled as limited, scarce, or rare will likely be more attractive to *convenience shoppers. Store-oriented shoppers* were most highly influenced by *consensus*, which suggests that, when in doubt, *convenience shoppers* look to others in their social circle for what to buy. This implies that by highlighting the products that others in their social circles have purchased, the shopping decision of *convenience shoppers* can be influenced. *Variety seekers*, on the other hand, were most highly affected by the influence strategy *authority*. This suggests that *variety seekers* can be influenced to purchase products because of people in authority.

These results suggest guidelines for the implementation of persuasive strategies by e-commerce platforms to make these persuasive strategies more effective in influencing the purchasing decisions of shoppers. For example, in a bid to make people shop for more healthful foods when shopping online, an e-commerce platform can present healthful foods that are limited in edition, rare, or scarce to *convenience shoppers* because this category of shoppers is influenced to purchase products that are limited, rare, or scarce.

Although we are limited by a small sample size, we chose to use PLS-SEM in our study because PLS-SEM performs well even with small samples. We are still in the process of data collection and will repeat the study with more participants in the future. In addition, we will implement and test these results on an online shopping site in the future. In the proposed study, the strategies identified will be implemented for the different shopper types and the reactions of shoppers to these strategies will be noted and compared to the results presented in this study.

## Data Availability Statement

The datasets generated for this study are available on request to the corresponding author.

## Ethics Statement

The studies involving human participants were reviewed and approved by University of Saskatchewan Human Ethics Review Board. The patients/participants provided their informed consent to participate in this study.

## Author Contributions

All authors listed have made a substantial, direct and intellectual contribution to the work, and approved it for publication.

## Conflict of Interest

The authors declare that the research was conducted in the absence of any commercial or financial relationships that could be construed as a potential conflict of interest.

## Footnotes

^1^*Exploiting the Power of Reciprocity*: Available online at: https://medium.com/@Omri_Yacubovich/exploiting-the-power-of-reciprocity-e214f96147c

^2^https://www.smartpls.com/

^3^https://www.amazon.ca/

^4^https://www.laura.ca/

^5^https://www.walmart.ca/
